# A new innovative automated root canal device for syringe needle irrigation

**DOI:** 10.1016/j.jtumed.2021.07.011

**Published:** 2021-08-19

**Authors:** Kavalipurapu V. Teja, Sindhu Ramesh, Kaligotla A. Vasundhara, K.C. Janani, Jerry Jose, Gopi Battineni

**Affiliations:** a Department of Conservative Dentistry & Endodontics, Saveetha Dental College & Hospitals, Saveetha Institute of Medical & Technical Sciences, Saveetha University, Chennai, Tamilnadu, India; b Private Practitioner, Department of Prosthodontics & Implantology, Andhra Pradesh, India; c Telemedicine & Telepharmacy Department, School Medicinal and Health Product Sciences, University of Camerino, Camerino, Italy

Dear editor,

Irrigation of the root canal system is an essential aspect of endodontic treatment. Although recent irrigation agitation techniques, such as ultrasonic-assisted irrigation, have enhanced irrigant-wall interactions,^1^ the syringe needle-based delivery system remains a primary delivery system that is frequently employed, especially during the preparatory phase of the root canal. Computational fluid dynamic analysis-based reports have shown that various parameters^3^, ^4^, ^5^, ^6^, ^7^, ^8^, ^9^ such as velocity magnitude, turbulence intensity, and irrigant flow play major role in an efficient dynamic irrigation process. Nevertheless, in a clinical scenario, dynamic forces should never exceed apical physiological pressures developed.

Considering this fact, the optimal flow rates decided were 4–6 ml/min based on studies conducted using periapical pressure assessment models.^2^ Clinically, an operator cannot maintain the optimal flow rate constant, and there is no specific standardisation for syringe-needle-based irrigation systems. In particular, excessive forces develop at the thumb and wrist, leading to faster fatigue of the operator using needles with a thinner diameter for root canal irrigation. Boutskioskis et al.^10^ evaluated existing differences in irrigant flow rate, intra-barrel pressure, duration of irrigation and volume of irrigant delivered through various needle and stated that such manual irrigation varies according to multiple factors such as operator experience, gender, and barrel used, and indicated the difficulty of its standardisation in the clinical scenario. Therefore, irrigation using a manual syringe needle is a lacuna in the endodontic field. Hence, the present article discusses the working model of an automated irrigation device and its importance in the current scenario.

To overcome the difficulties encountered during manual syringe needle irrigation, we attempted to modify a syringe infusion pump as a novel automated irrigation device (Figure 1). (Indian Patent Copyright Application Number 201941037185). The syringe infusion pump (Acura S1 BPL Medical Technologies, Bangalore, India) is generally used for critical care and general infusion applications. The device has a horizontal and vertical fixation clamp (Figure 1) compatible with standard 5–60 ml syringes. It has an LED display with programmable infusion modes based on the rate and time of delivery of the drug.

**Figure 1:**
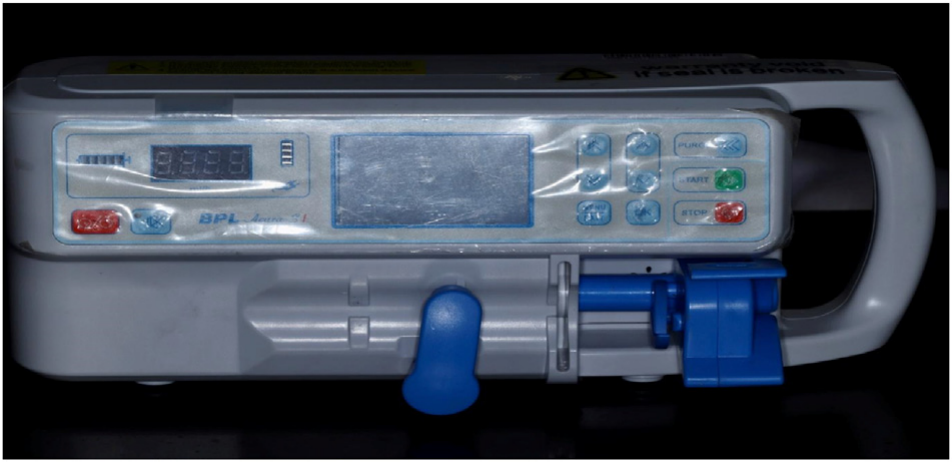
Depicting the syringe infusion pump (Acura S1 BPL Medical Technologies; Bangalore; India).

We modified the current device and connected it to a modified IV set tube (Figure 2) to simulate an irrigation system. The primary working mechanism of the proposed device is based on the rate delivery mode. We depended on the rate mode to deliver the irrigant at a specified rate. The rate modes possible in the current modified device ranged from 50 ml/h to 1200 ml/h. Thus, the converted rate modes possible were 0.5–12 ml/min.

**Figure 2:**
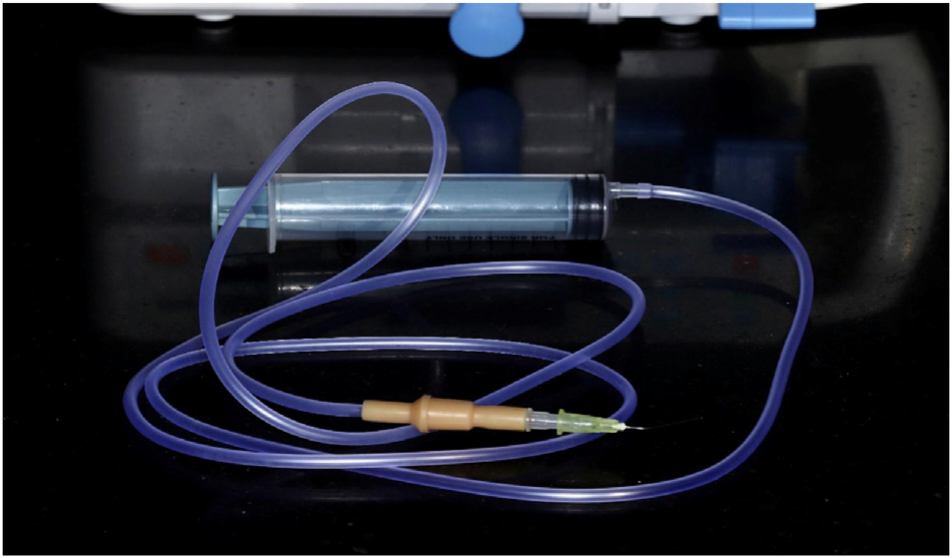
Depicting the customised modified IV set tube.

The device has an on/off sensor. When the device is switched on, the fixation slots can be adjusted to accommodate the syringe barrel. Once the syringe filled with irrigant is fixed, the sensors can be adjusted for the rate mode set at different values ranging from 0.5 to 12 ml/min to start the infusion (Figure 3). The device in the operating mode with the irrigant delivery is depicted in Figure 4.

**Figure 3:**
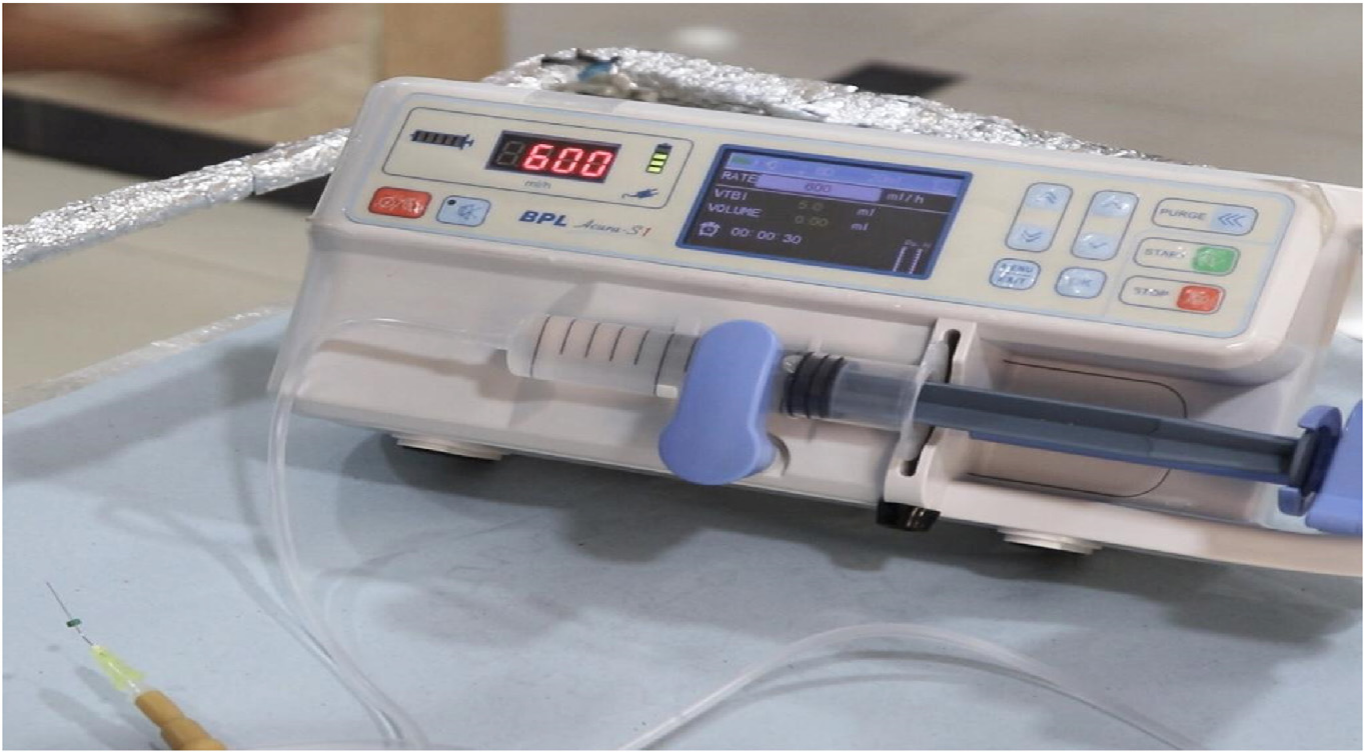
Depicting the working model of the device.

**Figure 4:**
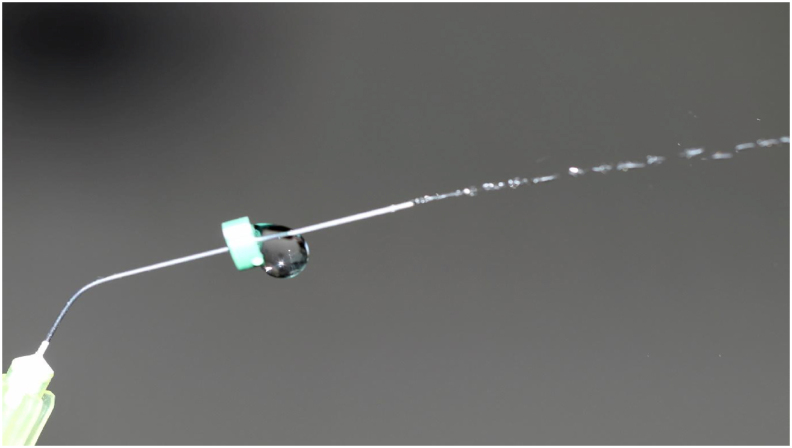
Figure shows the delivery of irrigant from a 30 Gauge side vented close ended needle at the rate of 6 ml/min.

Although root canal irrigation efficiency is primarily dependent on the dynamic forces during the flow, the generated apical pressures are the dictating factors for safe root canal irrigation in a clinical scenario. Therefore, the dynamic forces during root canal irrigation should never cross the physical and physiological limits.^2^ As the current device is primarily based on the controlled delivery of liquid, the actual apical pressure of irrigation would be minimal unless the needle is intentionally bound in the root canal.

Based on our experimental trials, the reported minimum and maximum flow rates possible with the device are around 0.5 ml/min and 12 ml/min, respectively. As the device can deliver the irrigant at constant and least possible flow rates, with the least apical pressures, it could be potentially beneficial in treating revascularization cases, preventing extrusion of the irrigant. The device is designed to be customised, and the tips used for routine root canal irrigation can be disposed after use to ensure that each tip is used only for one patient. This avoids issues regarding sterilisation. The device requires separate syringes or cartridges filled with different irrigants such as ethylenediaminetetraacetic acid (EDTA), chlorhexidine (CHX), and saline. Therefore, cartridge replacement can be performed after the use of each irrigant. The device has customised tips that are compatible with various needle types and designs. Therefore, as the time and the amount of liquid flow are adjustable in the device, they can be customised by an operator for a specific case scenario, preventing operator fatigue. When the current root canal irrigation strategy is compared, there is no specific standardisation for manual syringe needle irrigation in endodontics. In particular, for a clinician, it would be tedious to maintain continuous irrigant flow in the clinical (routine) scenario. Usually, it would be tedious to carry out syringe needle irrigation, with a constant and efficient flow, when using the finer-diameter needles. Hence, our irrigation device could reduce operator fatigue encountered during routine syringe needle irrigation.

Efficient irrigation mainly depends on the irrigant penetration at the apical one-third,^11^ both during manual and machine-assisted root canal irrigation. Hence, our innovation would improve irrigant penetration, especially at the apical one-third, for efficient syringe needle irrigation. However, no study has compared the present novel automated irrigation device with machine-assisted devices. Previous studies had focused on assessing machine-assisted devices such as Endobrush (C&S Micro instruments Ltd, Markham, Ontario, Canada), Easy clean irrigation device (Easy Dental Equipment, Belo Horizonte, MG, Brazil), and Quantec-E irrigation system (SybronEndo, Orange, CA).^12^ Usually, Endobrush and Easy clean devices are used after root canal preparation, but the Quantec-E device is used during the instrumentation phase as a continuous irrigant delivery system. However, the device currently described in the present article is mainly a modified and automated version of manual syringe needle irrigation, which is different from other machine-assisted irrigation devices. Most of the research has concentrated on assessing the devices for the removal of dentinal debris and root canal cleanliness.^12^ These automated devices work better in cases with anatomical root canal complexities such as fins, cul-de-sacs, isthmus, and in cases with irregular anatomies such as C-shaped canals and oval root canal anatomies. The major disadvantage in comparing these devices for clinical use is mainly due to the lack of evidence in the extensive literature on the use of these devices in patients.

Endo brushes are adjunctive devices for the debridement of root canal walls and agitation of root canal irrigants and may be indirectly involved in the transfer of irrigants within the root canal spaces.^12^ The major disadvantage of Endobrush (C&S Micro instruments Ltd, Markham, Ontario, Canada) is its size, which cannot be used to its full working length, which eventually results in debris packing in the apical region of the canal. Easy clean irrigation device (Easy Dental Equipment, Belo Horizonte, MG, Brazil) is a #25/0.04 plastic instrument with a reciprocating motion connected to an electric motor. Cesario et al. reported that the Easy Clean group achieved similar outcomes as the conventional irrigation approaches when used in reciprocating action.^13^ The Quantec-E irrigation system (SybronEndo, Orange, CA) is a continuous irrigation rotary instrumentation device. A previous study reported no significant difference in canal cleanliness in both the middle and apical thirds when compared with conventional syringe irrigation.^14^

Currently, manual syringe-based irrigation is unstandardized, jeopardising the treatment. However, our automated device delivers irrigants at constant flow rates, improvising flow, and apical pressures. Although the recent technology in endodontics overcomes the disadvantages of syringe needle irrigation, it is still a primary mode of delivery system during the preparatory phases of root canal treatment. Our automated irrigation device would help clinicians achieve efficient syringe needle irrigation with minimal or no fatigue as it is automated, and the operator merely has to irrigate efficiently during root canal shaping.

Previous studies have been conducted at the in vitro and in vivo levels (unpublished) using this novel automated irrigation device. Our unpublished study results showed constant recorded irrigant flow rates, with less operator fatigue even after using different needles, gauges, and barrels with automated irrigation devices. Therefore, prior pre-clinical assessments were carried out before patient experimentation. Currently, we are working on using the device on patients using a standard randomised controlled trial design that compares it with an automated irrigation device. However, the device is still in the preliminary phase of development, and several modifications are needed to improve the present apparatus for clinical applicability. The present modification will surely be a game-changer for current irrigation technology and would aid further developments that ensure easier and more efficient syringe needle irrigation.

## Source of funding

This research did not receive any specific grant from funding agencies in the public, commercial, or not-for-profit sectors.

## Conflict of interest

The authors have no conflict of interest to declare.

## Ethical approval

The authors confirm that this letter was prepared in accordance with the COPE roles and regulations. Given the nature of the letter, an IRB review was not required.

## Authors' contributions

All authors contributed equally to this work. All authors critically reviewed and approved the final draft and are responsible for the content and similarity index of the manuscript. after reference 3.
